# Development and Validation of a Deep Learning Algorithm for Mortality Prediction in Selecting Patients With Dementia for Earlier Palliative Care Interventions

**DOI:** 10.1001/jamanetworkopen.2019.6972

**Published:** 2019-07-12

**Authors:** Liqin Wang, Long Sha, Joshua R. Lakin, Julie Bynum, David W. Bates, Pengyu Hong, Li Zhou

**Affiliations:** 1 Harvard Medical School, Boston, Massachusetts; 2 Division of General Internal Medicine and Primary Care, Brigham and Women’s Hospital, Boston, Massachusetts; 3 Michtom School of Computer Science, Brandeis University, Waltham, Massachusetts; 4 Department of Psychosocial Oncology and Palliative Care, Dana-Farber Cancer Institute, Boston, Massachusetts; 5 Division of Palliative Medicine, Brigham and Women’s Hospital, Boston, Massachusetts; 6 Division of Geriatrics and Palliative Care, Department of Medicine, University of Michigan School of Medicine, Ann Arbor

## Abstract

**Importance:**

Early palliative care interventions drive high-value care but currently are underused. Health care professionals face challenges in identifying patients who may benefit from palliative care.

**Objective:**

To develop a deep learning algorithm using longitudinal electronic health records to predict mortality risk as a proxy indicator for identifying patients with dementia who may benefit from palliative care.

**Design, Setting, and Participants:**

In this retrospective cohort study, 6-month, 1-year, and 2-year mortality prediction models with recurrent neural networks used patient demographic information and topics generated from clinical notes within Partners HealthCare System, an integrated health care delivery system in Boston, Massachusetts. This study included 26 921 adult patients with dementia who visited the health care system from January 1, 2011, through December 31, 2017. The models were trained using a data set of 24 229 patients and validated using another data set of 2692 patients. Data were analyzed from September 18, 2018, to May 15, 2019.

**Main Outcomes and Measures:**

The area under the receiver operating characteristic curve (AUC) for 6-month and 1- and 2-year mortality prediction models and the factors contributing to the predictions.

**Results:**

The study cohort included 26 921 patients (16 263 women [60.4%]; mean [SD] age, 74.6 [13.5] years). For the 24 229 patients in the training data set, mean (SD) age was 74.8 (13.2) years and 14 632 (60.4%) were women. For the 2692 patients in the validation data set, mean (SD) age was 75.0 (12.6) years and 1631 (60.6%) were women. The 6-month model reached an AUC of 0.978 (95% CI, 0.977-0.978); the 1-year model, 0.956 (95% CI, 0.955-0.956); and the 2-year model, 0.943 (95% CI, 0.942-0.944). The top-ranked latent topics associated with 6-month and 1- and 2-year mortality in patients with dementia include palliative and end-of-life care, cognitive function, delirium, testing of cholesterol levels, cancer, pain, use of health care services, arthritis, nutritional status, skin care, family meeting, shock, respiratory failure, and swallowing function.

**Conclusions and Relevance:**

A deep learning algorithm based on patient demographic information and longitudinal clinical notes appeared to show promising results in predicting mortality among patients with dementia in different time frames. Further research is necessary to determine the feasibility of applying this algorithm in clinical settings for identifying unmet palliative care needs earlier.

## Key Points

**Question:**

How does a deep learning algorithm using patient demographic information and longitudinal clinical notes to predict mortality risk perform as a proxy indicator for identifying patients with dementia who need palliative care?

**Findings:**

In this cohort study, for a validation data set of 2692 adult patients with dementia, mortality prediction models reached an area under the receiver operating characteristic curve of 0.978 for predicting death in 6 months, 0.956 for 1 year, and 0.943 for 2 years.

**Meaning:**

Deep learning appears to show promising results in mortality risk stratification in patients with dementia.

## Teaser

This cohort study develops and validates a deep learning algorithm using longitudinal electronic health records to predict mortality risk as a proxy indicator for identifying patients with dementia who may benefit from palliative care.

## Introduction

A growing number of US adults have Alzheimer disease and related dementias (ADRD).^1,2,3^ As dementia progresses, patients frequently receive interventions that can add to this burden,^4,5^ including tube feeding^6,7^ and hospital transfers.^8^ These treatments, if unhelpful in achieving patient and family goals, can potentially contribute to poor quality of life and family dissatisfaction,^9^ while also driving higher health care expenditures at the end of life.^10,11,12^ Early palliative care interventions hold promise in the population with ADRD,^13,14^ because the delivery of palliative care improves patient care and family bereavement outcomes and results in more appropriate use of health care resources in other patient populations.^15,16,17,18,19,20^ As such, national organizations are intensifying calls for increasing the reach of palliative care to more patients.^21,22^ However, knowing which patients may benefit from palliative care and when is difficult and remains a key barrier to expanding reach. Data suggest that patients with ADRD receive palliative care late in life, possibly interfering with accrual of benefit to patients and families.^23^ A predictive tool improving the timeliness of palliative care interventions in patients with ADRD could help to optimally target scarce resources and improve patient care.

Current approaches to identification of patients with palliative care needs rely heavily on busy health care professionals, claims data, and logistic regression models, each of which has inherent limitations.^24,25,26^ Several survival prediction tools, such as the Palliative Performance Scale^27^ and Palliative Prognostic Score,^28^ have been developed for specialty palliative care or hospice applications and are based on exponential multiple regression analysis by considering expert-curated features such as functional ability, self-care, and oral intake. However, these tools are limited by requiring expert clinical opinions for each patient.^27,28^ Prior efforts to develop prognostic models to predict survival for larger groups of patients specifically with ADRD have been limited to specific clinical settings (eg, nursing homes^29^) or data sets (eg, caregiver interviews and claims records^30^). Although shorter-term prediction models, such as 6-month^29^ and 12-month^30,31^ predictions, are helpful for some palliative care applications (eg, hospice care), longer-term prediction models are also important in ADRD not only owing to the nature of the disease and associated cognitive and functional decline but also because many of the essential requirements of high-value palliative care, such as advance care planning, serious illness communications, and meaningful conversations about patients’ goals and values, must be performed earlier in the disease course.

To identify patients with ADRD who may benefit from earlier palliative care interventions, we developed and validated 6-month, 1-year, and 2-year mortality prediction models, with a primary focus on the 2-year model, using a deep neural network and longitudinal clinical notes from electronic health records (EHRs). We also improved the transparency and interpretability of complex machine learning predictive models by determining the predictive factors derived from clinical notes associated with mortality in dementia populations.

## Methods

### Clinical Setting and Data Sources

This retrospective cohort study was conducted at Partners HealthCare System (PHS), a nonprofit integrated health care system in Boston, Massachusetts. The PHS care delivery network was founded by 2 academic medical centers (Brigham and Women’s Hospital and Massachusetts General Hospital) and includes multiple community hospitals, specialty facilities, community health centers, and other health-related entities (such as a rehabilitation hospital). We collected data from the PHS Research Patient Data Registry, a clinical data registry that gathers medical records from various hospital systems, and the Enterprise Data Warehouse (EDW), which stores patients’ EHR data. The Massachusetts Death Index was obtained to supplement death data available in PHS data sets. This study was approved by the institutional review board of PHS with waiver of informed consent from study participants for secondary use of electronic health records. This study followed the Strengthening the Reporting of Observational Studies in Epidemiology (STROBE) reporting guideline for cohort studies.

### Study Cohort

We identified a cohort of study patients older than 18 years with ADRD who visited PHS from January 1, 2011, through December 31, 2017, using *International Classification of Diseases, Ninth Revision, Clinical Modification* codes 290, 294.1, 294.2, 331.0, 331.1, 331.2, and 331.82 and *International Statistical Classification of Diseases, Tenth Revision, Clinical Modification* codes F00 to F03, G30.0, G30.1, G30.8, G30.9, G31.0, G31.1, G31.83, and G31.9 (see the fully expanded list of codes and their descriptions in eTable 1 in the Supplement). We further restricted the cohort to those patients who (1) were known to be deceased or had clinical notes available within 2 years before the date of their last visit in our health care system and (2) had more than 1 documented clinical note.

### Data Preparation

In this study, we included patient age, sex, race, ethnicity, educational level, and marital status, all of which were reported by participants or proxy respondents (such as a family caregiver) and collected in the EHR as part of regular clinical care. We obtained all types of clinical notes (eg, clinic visit notes, discharge summaries, and consultation notes) except narrative clinical reports (eg, radiology and pathology reports) documented during the study period. We further aggregated clinical notes by date, concatenating all notes documented on the same date into 1 note event in the data set. Thereafter, we used a natural language processing approach similar to the one developed in a previous study^23^ and generated 500 latent topics from clinical notes as well as topic document proportion scores (indicating the proportion of a document containing information about the topic) for each note event.

To prepare a labeled data set of the study cohort for predictive modeling, we obtained the vital status (alive or deceased) of the study cohort from Partners’ Enterprise Data Warehouse and the Massachusetts Death Index. We linked our study cohort to the Massachusetts Death Index to obtain additional death information by exact matching on a combination of Social Security number, sex, and date of birth or a combination of patient name, sex, date of birth, and city of residence. We also retrieved patients’ date of last visit to a PHS-affiliated health care facility as of September 18, 2018, to determine the vital status of the study cohort.

### Development of the Models

**Figure 1.**
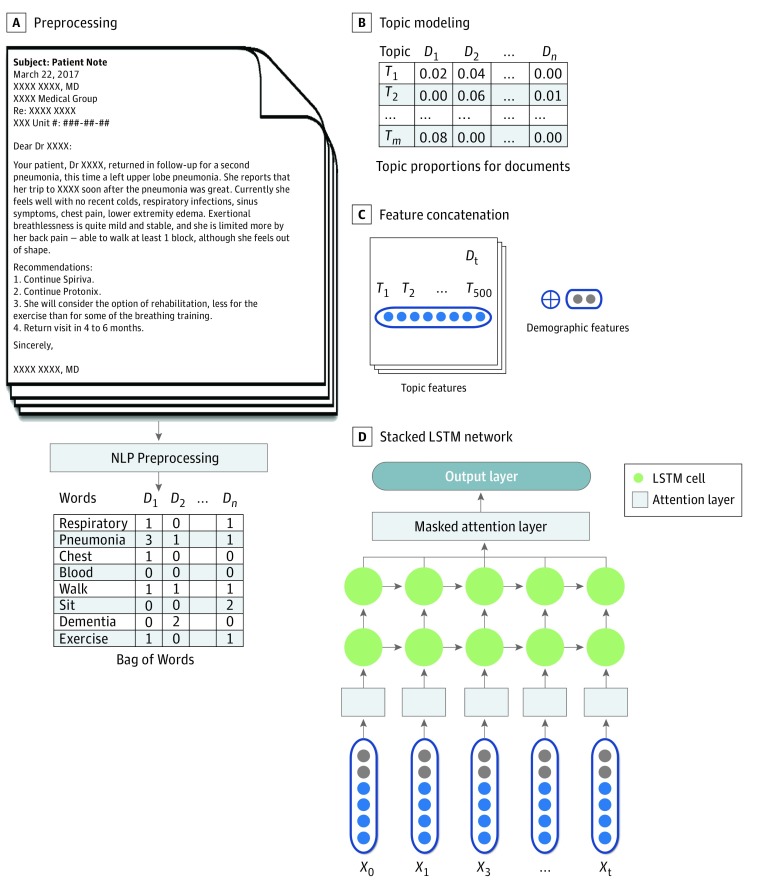
Overview of the Predictive Modeling Using Longitudinal Clinical Notes and Demographics of Patients With Dementia A, Natural language processing (NLP) preprocessing to convert raw clinical text into a bag of words after removing punctuation and other symbols. D indicates document. B, Generation of the topic (T) features from clinical documents (D) using topic modeling. C, Concatenation of the topic features and the demographic features to form input to the neural network. D, A stacked long short-term memory (LSTM) neural network with 2 attention layers (boxes marked in gray). X indicates input variables of the neural network, which were also the results from step C.

We formulated our mortality prediction as a classification task in which the model aimed to make binary predictions at a time of a specific note event, namely whether the patient was going to die in 6 months or 1 or 2 years. Deep learning is a process of training deep neural networks to perform such a classification task. We chose the long short-term memory (LSTM) network,^32^ a novel recurrent neural network in conjunction with an appropriate gradient-based learning algorithm, as the network architecture in our algorithm, given LSTM’s ability to model longitudinal EHR data. Two prior studies^33,34^ have used LSTM networks for predicting in-hospital mortality, postdischarge mortality, and 30-day readmissions. The deep learning neural network we constructed is composed of 2 stacked LSTM layers with 2 attention layers: one placed between the input layer and the LSTM layers and the other between the LSTM layers and the output layers (Figure 1). The inputs of our deep neural network model were the variables concatenated with topic document proportion scores of 500 topics and patient demographic variables. The stacked LSTM layers supported a hierarchical abstraction of the input data. The attention layers were used to improve model performance as well as trace the importance of temporal inputs while the model was making predictions.^35^ Thus, we were able to extract a weight for each feature at a specific note event representing its importance to the model’s prediction. These weights were used to rank the predictive power of the topic features with respect to the 6-month, 1-year, and 2-year mortality predictions.

We randomly split the study cohort into development and validation data sets with a ratio of 9:1. The development data set was further divided into training and tuning parts, with 89% of the data set used to optimize the weights of the neural network according to cross-entropy loss function and 11% used to optimize hyperparameters (eg, learning rates, depth of the network, size of the hidden layers). We set the minimum required number of note events to 2 so that our model would have a sufficient medical history for each patient to make reliable predictions. Any patients having fewer than 2 note events were excluded from the training and validation data sets. We tuned other hyperparameters using a grid search for achieving an optimal performance in the tuning data set. In addition, we used dropout to avoid model overfitting, a method that shuts down a random percentage of artificial neurons during each training epoch to reduce interdependent learning among the neurons in the model and to force the model to learn more robust internal representations.

### Statistical Analysis

Data were analyzed from September 18, 2018, to May 15, 2019. We validated the final 6-month, 1-year, and 2-year mortality prediction models using the validation data set and reported the performance of our approach using the area under the receiver operating characteristics curve (AUC). The 95% CIs were computed with 2000 stratified bootstrap replicates.^36^ All statistical analyses were performed using R software, version 3.5.3 (R Foundation for Statistical Computing).^37^

## Results

We present patient demographic information and note event characteristics in Table 1. The study cohort included 26 921 patients (16 263 women [60.4%] and 10 658 men [39.6%] men; mean [SD] age, 74.6 [13.5] years) who met the inclusion criteria. Of those patients, we reserved 10.0% (n = 2692) for validation, leaving the remainder (n = 24 229) for the development of the models. Of the 24 229 patients in the development set, 14 632 (60.4%) were women and 9597 (39.6%) were men, and the mean (SD) age was 74.8 (13.2) years. Of the 2692 patients in the validation set, 1631 (60.6%) were women and 1061 (39.4%) were men, and the mean (SD) age was 75.0 (12.6) years. Among these 2 data sets, 23 039 patients were white (85.6%), 24 661 were non-Hispanic (91.6%), and 12 385 (46.0%) died from January 1, 2011, through September 18, 2018. We labeled a total of 959 628 note events with 6-month, 1-year, and 2-year mortality. A mean of 35 to 36 note events were found per person. More than 75% of the note events were documented more than 2 years before death or last patient visit to PHS facilities. In addition, more notes were documented nearer to death (eg, 47 219 note events 0-3 months before death vs 28 472 note events 4-6 months before death) in the development data set.

**Table 1.** Characteristics of the Study Cohort and Note Events

Characteristic	Data Set^a^	
	Development (n = 24 229)	Validation (n = 2692)
Age, mean (SD), y^b^	74.8 (13.2)	75.0 (12.6)
Died^c^	11 138 (46.0)	1247 (46.3)
Female sex	14 628 (60.4)	1631 (60.6)
Race		
White	20 734 (85.6)	2305 (85.6)
Black	1302 (5.4)	159 (5.9)
Others	515 (2.1)	47 (1.7)
Unknown	1678 (6.9)	182 (6.8)
Ethnicity		
Non-Hispanic	22 190 (91.6)	2471 (91.8)
Hispanic	1400 (5.8)	147 (5.5)
Unknown	639 (2.6)	74 (2.7)
Marital status		
Married or partnered	10 490 (43.3)	1162 (43.2)
Single, divorced, or widowed	12 324 (50.9)	1378 (51.2)
Unknown	1415 (5.8)	152 (5.6)
Educational level		
College and above	6955 (28.7)	755 (28.0)
High school or equivalent	7392 (30.5)	812 (30.2)
Did not complete high school	2181 (9.0)	239 (8.9)
Unknown	7701 (31.8)	886 (32.9)
No. of total note events^d^	863 160	96 468
No. of note events per patient, mean (SD)	35.6 (49.2)	38.8 (49.8)
No. of note events in time before death, mo		
0-3	47 219 (5.5)	5238 (5.4)
4-6	28 472 (3.3)	3223 (3.3)
7-12	49 709 (5.8)	5684 (5.9)
13-24	82 767 (9.6)	9465 (9.8)
≥25^e^	654 993 (75.9)	72 858 (75.5)

^a^ Unless otherwise indicated, data are expressed as number (percentage) of patients. Percentages have been rounded and may not total 100.

^b^ Calculated at the beginning of the study period (ie, January 1, 2011).

^c^ Collected from January 1, 2011, through September 18, 2018.

^d^ The note events met the following inclusion criteria: (1) can be labeled in terms of 2-year mortality and (2) have more than 10 words after the natural language processing preprocessing.

^e^ A significant increase of note events documented more than 2 years before death was due to the inclusion of patients who were still living as of the most recent date of encounter recorded in the patient’s record in our health care system.

**Figure 2.**
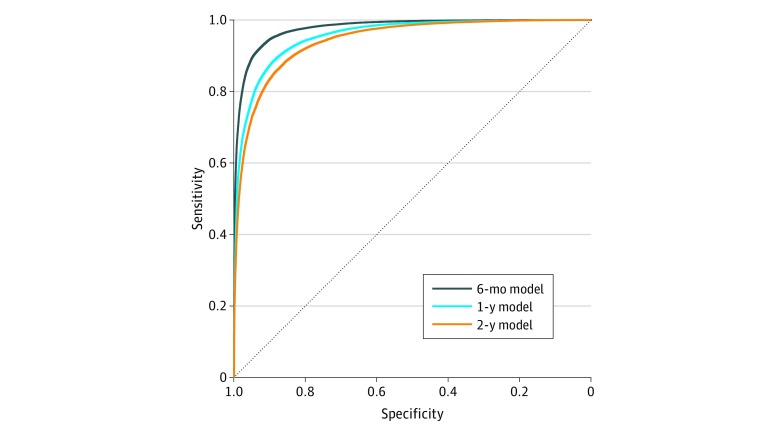
Receiver Operating Characteristic Curves of the Deep Learning Models in Predicting Patient Mortality In a validation data set of 2692 patients with Alzheimer disease and related dementia, the deep learning–based models showed high note events–level classification of 6-month, 1-year, and 2-year mortality, achieving areas under the receiver operating characteristic curve of 0.978 (95% CI, 0.977-0.978) for the 6-month model, 0.956 (95% CI, 0.955-0.956) for the 1-year model, and 0.943 (95% CI, 0.942-0.944) for the 2-year model.

The trained models made predictions at the time stamps of all the note events of the patients in the validation data set. By checking the classification of each prediction event against patient vital status, our proposed model reached an AUC of 0.943 (95% CI, 0.942-0.944) for predicting 2-year mortality, an AUC of 0.956 (95% CI, 0.955-0.956) for predicting 1-year mortality, and an AUC of 0.978 (95% CI, 0.977-0.978) for predicting 6-month mortality (Figure 2).

**Figure 3.**
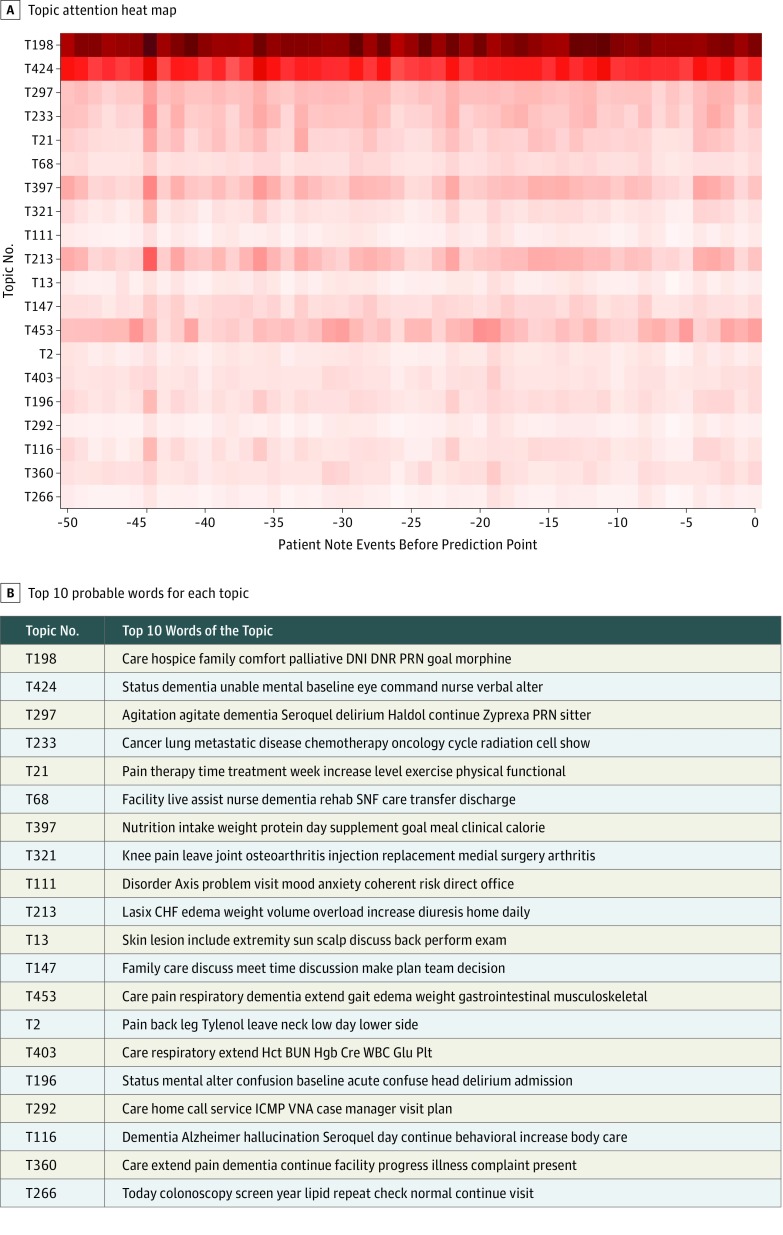
Topic Attention Heatmap and Corresponding Note Events Predicting 2-Year Mortality A, Topic attention heatmap showing, in predicting 2-year mortality at the time stamp of the last note event, the contribution of selected 20 predictive topics from prior 50 note events. B, The topic numbers and their top 10 probable words. BUN indicates blood urea nitrogen; CHF, congestive heart failure; Cre, creatinine; DNI, do not intubate; DNR, do not resuscitate; Glu, glucose; Hct, hematocrit; Hgb, hemoglobin; ICMP, intensive care management program; Plt, platelet; PRN, prescription as needed; SNF, skilled nursing facility; VNA, Visiting Nurse Association; and WBC, white blood count.

At the patient level, the weights of the topics extracted from the attention layer during the prediction were informative of which topics at which prior note event were predictive (Figure 3). By summing the weights of each topic in making predictions at all note events of the 2692 patients in the validation cohort, we identified a list of topics as top-ranked predictive features for 6-month, 1-year, and 2-year mortality. The top-ranked latent topics associated with 6-month and 1- and 2-year mortality in patients with dementia include palliative and end-of-life care, cognitive function, delirium, testing of cholesterol levels, cancer, pain, use of health care services, arthritis, nutritional status, skin care, family meeting, shock, respiratory failure, and swallowing function. Table 2 shows the top 20 ranked topics associated with 6-month, 1-year, and 2-year mortality as well as their labels annotated by one of us (J.R.L.); the top 100 ranked topics are available in eTables 2 to 4 in the Supplement.

**Table 2.** Top 20 Predictive Topics Associated With 6-Month, 1-Year, and 2-Year Mortality

Rank	Manual Label	Top 15 Probable Words
**Top-Ranked Predictive Topics for 2-Year Model**		
1	Palliative and end-of-life care	Care hospice family comfort palliative DNI DNR PRN goal morphine CMO discussion dementia measure pain
2	Cognitive function	Status dementia unable mental baseline eye command nurse verbal alter hypernatremia open due poor lethargy
3	Cholesterol level testing	Cholesterol LDL result test total blood compare HDL bad normal function triglyceride good hemoglobin medical
4	Delirium	Agitation agitate dementia Seroquel delirium Haldol continue Zyprexa PRN sitter behavior psych time medication trazodone
5	Laboratory testing	Range normal detail test blood result function check glucose creatinine potassium kidney total calcium BUN
6	Cancer	Cancer lung metastatic disease chemotherapy oncology cycle radiation cell show chemotherapy tumor carcinoma mass adenocarcinoma
7	Pain evaluation and treatment	Pain therapy time treatment week increase level exercise physical functional report activity tissue visit hip
8	Hospital care	Date information case phone admit information referral status hospital Salem care bed gender contact page
9	Results communication	Result test letter question dear receive contact manager share normal hesitate blood function show report
10	Facility care	Facility live assist nurse dementia rehabilitation SNF care transfer discharge staff term fall ALF long
11	Nutritional status	Nutrition intake weight protein day supplement goal meal clinical calorie kcal daily Ensure diet continue
12	Spanish documentation^a^	Los para una con usted por tiene puede sus del medico dolor como medicamentos sobre
13	Health care encounter	Hospital general medication Massachusetts management medicine associate fax internal phone pharmacy electronically transmit prescription prepare
14	Arthritis	Knee pain leave joint osteoarthritis injection replacement medial surgery arthritis effusion total lateral bilateral motion
15	Mental status examination	Disorder Axis problem visit mood anxiety coherent risk direct office treatment current pain exam status
16	Heart failure	Lasix CHF edema weight volume overload increase diuresis home daily heart failure SOB admission fluid
17	Skin care	Skin lesion include extremity sun scalp discuss back perform exam papule upper dermatology face nevus
18	Family meeting	Family care discuss meet time discussion make plan team decision son discus understand medical risk
19	General medical care	Care pain respiratory dementia extend gait edema weight gastrointestinal musculoskeletal med review wheeze clear erythema
20	Pain evaluation and treatment	Pain back leg Tylenol leave neck low day lower side muscle tenderness week worse ibuprofen
**Top-Ranked Predictive Topics for 1-Year Model**		
1	Palliative and end-of-life care	Care hospice family comfort palliative DNI DNR PRN goal morphine CMO discussion dementia measure pain
2	Cognitive function	Status dementia unable mental baseline eye command nurse verbal alter hypernatremia open due poor lethargy
3	Laboratory testing	Range normal detail test blood result function check glucose creatinine potassium kidney total calcium BUN
4	Cholesterol level testing^b^	Cholesterol LDL result test total blood compare HDL bad normal function triglyceride good hemoglobin medical
5	Results communication	Result test letter question dear receive contact manager share normal hesitate blood function show report
6	Medication delivery	Tablet day tablet BID capsule QHS PRN direct acid HCL unit release vitamin visit TID
7	Family meeting	Family care discuss meet time discussion make plan team decision son discus understand medical risk
8	Physical examination	Normal time note sit review pulse status history interpretation inspection pain physician resp skin respiratory
9	Delirium	Agitation agitate dementia Seroquel delirium Haldol continue Zyprexa PRN sitter behavior psych time medication trazodone
10	Health care encounter	Hospital general medication Massachusetts management medicine associate fax internal phone pharmacy electronically transmit prescription prepare
11	Facility care	Facility live assist nurse dementia rehabilitation SNF care transfer discharge staff term fall ALF long
12	Cholesterol level testing^b^	Test cholesterol blood follow function laboratory phone recent normal office dear range medicine Parkman kidney
13	Pain evaluation and treatment	Pain therapy time treatment week increase level exercise physical functional report activity tissue visit hip
14	Spanish documentation^a^	Los para una con usted por tiene puede sus del mdico dolor como medicamentos sobre
15	Hospital care	Date information case phone admit information referral status hospital Salem care bed gender contact page
16	Nursing care	Continue progress rate output intake hour today SPO urine monitor total overnight shift nurse event
17	Mental status examination	Disorder axis problem visit mood anxiety coherent risk direct office treatment current pain exam status
18	Swallowing function	Liquid swallow diet aspiration dysphagia thick puree SLP nectar solid thin speech continue soft consistency
19	Cancer	Cancer lung metastatic disease chemotherapy oncology cycle radiation cell show chemotherapy tumor carcinoma mass adenocarcinoma
20	Nutritional status	Nutrition intake weight protein day supplement goal meal clinical calorie kcal daily ensure diet continue
**Top-Ranked Predictive Topics for 6-Month Model**		
1	Palliative and end-of-life care	Care hospice family comfort palliative DNI DNR PRN goal morphine CMO discussion dementia measure pain
2	Cognitive function	Status dementia unable mental baseline eye command nurse verbal alter hypernatremia open due poor lethargy
3	Laboratory testing	Range normal detail test blood result function check glucose creatinine potassium kidney total calcium BUN
4	Cholesterol level testing	Cholesterol LDL result test total blood compare HDL bad normal function triglyceride good hemoglobin medical
5	Results communication	Result test letter question dear receive contact manager share normal hesitate blood function show report
6	Physical examination	Normal time note sit review pulse status history interpretation inspection pain physician resp skin respiratory
7	Family meeting	Family care discuss meet time discussion make plan team decision son discuss understand medical risk
8	Healthcare encounter	Hospital general medication Massachusetts management medicine associate fax internal phone pharmacy electronically transmit prescription prepare
9	Delirium	Agitation agitate dementia Seroquel delirium Haldol continue Zyprexa PRN sitter behavior psych time medication trazodone
10	Nutritional status	Nutrition intake weight protein day supplement goal meal clinical calorie kcal daily Ensure diet continue
11	Hospital care	Progress hospitalization adult absence risk continue pediatric fall actual sign discharge infection symptom condition pressure
12	Respiratory failure	Respiratory BIPAP failure pulmonary oxygen hypoxia sit ICU edema status transfer Lasix distress require improve
13	Shock	Shock sepsis transfer ICU MICU hypotension septic failure set pressor continue fluid require improve respiratory
14	Swallowing function	Swallow SLP liquid aspiration oral speech thin dysphagia solid diet puree language cough consistency thick
15	Swallowing function	Liquid swallow diet aspiration dysphagia thick puree SLP nectar solid thin speech continue soft consistency
16	Physical examination	Pressure blood normal edema pulse weight clear chest murmur today year regular daily heart extremity
17	Medication delivery	Tablet day tablet BID capsule QHS PRN direct acid HCL unit release vitamin visit TID
18	Intensive care	Intubate vent airway goal day continue tube Fio CMH respiratory care line rate ICU ETT
19	Facility care	Facility live assist nurse dementia rehabilitation SNF care transfer discharge staff term fall ALF long
20	General medical care	Care pain respiratory dementia extend gait edema weight gastrointestinal musculoskeletal med review wheeze clear erythema

Abbreviations (approximated from clinical notes): ALF, assisted living facility; BID, twice daily; BIPAP, bilevel positive airway pressure; BUN, blood urea nitrogen; CHF, congestive heart failure; CMH, centimeters of water; CMO, comfort measures only; DNI, do not intubate; DNR, do not resuscitate; ETT, endotracheal tube; Fio, fraction of inhaled oxygen; HCL, hydrochloride; HDL, high-density lipoprotein; ICU, intensive care unit; kcal, kilocalorie; LDL, low-density lipoprotein; MICU, medical ICU; PRN, prescription as needed; psych, psychology or psychiatry or some variation on these terms; QHS, at bedtime; resp, respiratory or respiration or some similar variations; SLP, speech-language pathology; SNF, skilled nursing facility; SOB, shortness of breath; SPO, peripheral capillary oxygen saturation; TID, three times daily.

^a^ This topic groups common words in Spanish because of the inclusion of clinical notes written in Spanish, primarily among the notes for communication with patients, including patient letters and instructions.

^b^ Topics with similar words were labeled with the same name.

## Discussion

This study demonstrates that a deep neural network trained using a large data set with patient demographics and longitudinal clinical notes from the EHR can be accurate and useful in predicting 6-month, 1-year, and 2-year mortality and thus could be used as a proxy for selecting patients who may benefit from palliative care assessment. The high performance (AUC scores) of all 3 models shows that clinical notes along with patient demographics are informative, and the deep learning neural network structure can successfully capture short- and long-range longitudinal patterns. In addition, converting clinical notes into clinically meaningful topics using topic modeling allows us to trace and visualize how the model made its prediction for each patient (Figure 3). Meanwhile, at the population level, the model helps us to identify what factors are strongly associated with mortality risk in different time frames in patients with ADRD (Table 2).

In the past, studies of mortality prediction have relied on claims data,^38^ administrative data,^39^ or other types of data (eg, surveys),^40^ but few have used clinical notes. We believe that this study is the first to investigate clinical notes in a deep neural network to identify topics associated with mortality prediction among patients with ADRD. In the LSTM-based neural network, clinical notes contribute to mortality prediction in 2 aspects: the longitudinal patterns of the documentation and the content of clinical notes. First, frequent documentation in the medical record likely indicates increasing severity of illness and worsening frailty in the context of ADRD; thus, with the help of the LSTM neural network, long- and short-term longitudinal patterns can be identified for mortality prediction. Second, topics generated using the topic modeling method captured semantic and syntactic structures of large quantities of clinical notes, providing rich information for mortality prediction. Among 500 topics, top-ranked predictive factors associated with 6-month, 1-year, and 2-year mortality include palliative and end-of-life care, cognitive function (eg, dementia status, delirium), laboratory testing (eg, testing of cholesterol levels), cancer, pain, use of health care services (eg, hospital or facility care, health care encounter, intensive care, and nursing care), arthritis, nutritional status, skin care, family meeting, result communication, swallowing function, shock, respiratory failure, and medication delivery, among others. Some of these topics indicate that health care professionals may recognize a patient’s decline (such as notation of palliative and end-of-life care), while others may signal changing patient conditions that health care professionals have yet to recognize (such as cognitive function, delirium, and functional status). Only a few studies explicitly list variables used to predict mortality in the ADRD population. For example, Mitchell et al^29,41^ included length of stay, dyspnea, pressure ulcers, total functional dependence, being bedbound most of the day, insufficient intake, bowel incontinence, body mass index, weight loss, and congestive heart failure as variables that best predict 6-month survival. Using the topic modeling method, we were able to capture topics that seem similar to variables selected a priori as well as additional variables that may not be available in many structured data.

Our models can be calculated with much less time and effort in large patient populations compared with existing screening methods (eg, the “surprise question” method).^24,42^ Previous studies demonstrated that health care professionals, although directionally generally correct, have trouble estimating the timing of death.^43,44^ Long-term predictions are generally more difficult for humans; this may also apply to the machine, because our mortality prediction models achieved slightly lower performance when the prediction time frames became longer. However, our 2-year model still reached a high AUC of 0.943. Therefore, using deep learning predictive models in patient stratification in clinical practice has notable promise for identifying patients with ADRD who are approaching their last 1 or 2 years of life.

Although mortality is not the only important factor contributing to assessment of need for palliative care, tools such as this algorithm may provide an important proxy that health care professionals and systems can use to consider patients for possible palliative care interventions. By adjusting the sensitivity and specificity along the receiver operating characteristic curve, deep learning–based tools may be used to decrease the burden on health care professionals by identifying a manageable denominator of patients for consideration for interventions according to available palliative care resources. They may also help guide prioritization of patients’ needs based on predicted probability of mortality in a certain time frame. Importantly, these models should not be used in the absence of input from health care professionals, because computer-predicted mortality alone is not a decisive indicator of palliative care needs; the benefit of palliative care depends on far more than risk of death (eg, individual preferences, functional and quality of life effects of serious illness, psychosocial and spiritual needs, and burden of illness on caregiving networks). In addition, we have chosen a longer time frame of 2 years to target driving earlier conversations about patients’ goals and values in ADRD and to focus on patient-centric conversations rather than system-centric decisions such as enrollment in hospice.

### Limitations

One major limitation of our study is that our models have not been validated using external data sets. Because of population diversity and clinical documentation variations among different health care systems,^45^ we suspect that models trained from the data of one health care system may require additional tuning to be adaptable to other systems or EHRs. Therefore, until a systematic validation is performed, including using a different data set to assess the models’ generalizability, these models should not be widely applied to other health care systems. Second, machine learning–based models developed using EHR data may be subject to bias because the EHR generally contains more medical information for sicker patients, and this decreases the generalizability of the models to non-EHR settings.^46,47^ Third, model-based screening can only make predictions at the times when notes are available for patients, requiring a minimum of 2 note events. This limitation may affect the model’s capability to make predictions for all patients with ADRD at any time. Fourth, the ranking of the predictive topics, which was generated based on the attention of the neural network during the prediction for the validation cohort, does not directly correlate to the proportion of notes or patients to whom these topics apply, and such rankings may be subject to change as the predictive cohort changes. Fifth, prediction of mortality is only one component of identifying patients who may benefit from palliative care, and future predictive modeling efforts should move beyond mortality prediction to work on identifying broader needs for populations of seriously ill patients, such as predicting functional decline and effects on quality of life.^48^

## Conclusions

In evaluating predictive models as proxies for identifying patients with ADRD for early palliative care interventions, a deep machine learning algorithm using patient demographic information and topics derived from longitudinal clinical notes appears to show promising results in predicting 6-month, 1-year, and 2-year mortality. Further research is necessary to determine the feasibility of applying this algorithm in the clinical setting for identifying unmet palliative care needs earlier in patients with dementia.

## Notes

Supplement. **eTable 1.** List of *ICD* Diagnosis Codes for Identifying Patients Who Have Alzheimer’s Disease and Related Dementias **eTable 2.** Top 100 Ranked Predictive Topics for Predicting 2-Year Mortality **eTable 3.** Top 100 Ranked Predictive Topics for Predicting 1-Year Mortality **eTable 4.** Top 100 Ranked Predictive Topics for Predicting 6-Month Mortality
