# Supplementary material for: Development and Validation of a Deep Learning Algorithm for Mortality Prediction in Selecting Patients With Dementia for Earlier Palliative Care Interventions
Source: JAMA Netw Open. 2019 Jul 12;2(7):e196972. doi: 10.1001/jamanetworkopen.2019.6972 (PMC6628612; doi:10.1001/jamanetworkopen.2019.6972)
Supplement: Supplement. — eTable 1. List of ICD Diagnosis Codes for Identifying Patients Who Have Alzheimer’s Disease and Related Dementias eTable 2. Top 100 Ranked Predictive Topics for Predicting 2-Year Mortality eTable 3. Top 100 Ranked Predictive Topics for Predicting 1-Year Mortality eTable 4. Top 100 Ranked Predictive Topics for Predicting 6-Month Mortality [file jamanetwopen-2-e196972-s001.pdf]

Supplementary Online Content

Wang L, Sha L, Lakin JR, et al. Development and validation of a deep learning algorithm for mortality prediction in selecting patients with dementia for earlier palliative care interventions. *JAMA Netw Open.* 2019;2(7):e196972. doi:10.1001/jamanetworkopen.2019.6972

eTable 1. List of *ICD* Diagnosis Codes for Identify Patients Who Have Alzheimer's Disease and Related Dementias
eTable 2. Top 100 Ranked Predictive Topics for Predicting 2-Year Mortality
eTable 3. Top 100 Ranked Predictive Topics for Predicting 1-Year Mortality
eTable 4. Top 100 Ranked Predictive Topics for Predicting 6-Month Mortality

This supplementary material has been provided by the authors to give readers additional information about their work.

eTable 1. List of *ICD* Diagnosis Codes for Identify Patients Who Have Alzheimer's Disease and Related Dementias

Codes	Descriptions
ICD 10 Codes	
F01	Vascular dementia
F01.5	Vascular dementia
F01.50	Vascular dementia without behavioral disturbance
F01.51	Vascular dementia with behavioral disturbance
F02	Dementia in other diseases classified elsewhere
F02.8	Dementia in other diseases classified elsewhere
F02.80	Dementia in other diseases classified elsewhere without behavioral disturbance
F02.81	Dementia in other diseases classified elsewhere with behavioral disturbance
F03	Unspecified dementia
F03.9	Unspecified dementia
F03.90	Unspecified dementia without behavioral disturbance
F03.91	Unspecified dementia with behavioral disturbance
G30	Alzheimer's disease
G30.0	Alzheimer's disease with early onset
G30.1	Alzheimer's disease with late onset
G30.8	Other Alzheimer's disease
G30.9	Alzheimer's disease, unspecified
G31.0	Frontotemporal dementia
G31.1	Senile degeneration of brain, not elsewhere classified
G31.83	Dementia with Lewy bodies
G31.9	Degenerative disease of nervous system, unspecified
ICD 9 Codes	
290	Dementias
290.0	Senile dementia, uncomplicated
290.1	Presenile dementia
290.10	Presenile dementia, uncomplicated
290.11	Presenile dementia with delirium
290.12	Presenile dementia with delusional features
290.13	Presenile dementia with depressive features
290.2	Senile dementia with delusional or depressive features
290.20	Senile dementia with delusional features
290.21	Senile dementia with depressive features
290.3	Senile dementia with delirium
290.4	Vascular dementia

© 2019 Wang L et al. *JAMA Network Open*

290.40	Vascular dementia, uncomplicated
290.41	Vascular dementia, with delirium
290.42	Vascular dementia, with delusions
290.43	Vascular dementia, with depressed mood
290.8	Other specified senile psychotic conditions
290.9	Unspecified senile psychotic condition
294.1	Dementia in conditions classified elsewhere
294.10	Dementia in conditions classified elsewhere without behavioral disturbance
294.11	Dementia in conditions classified elsewhere with behavioral disturbance
294.2	Dementia, unspecified
294.20	Dementia, unspecified, without behavioral disturbance
294.21	Dementia, unspecified, with behavioral disturbance
331.0	Alzheimer's disease
331.1	Frontotemporal dementia
331.11	Pick's disease
331.19	Other frontotemporal dementia
331.2	Senile degeneration of brain
331.82	Dementia with lewy bodies

© 2019 Wang L et al. *JAMA Network Open*

eTable 2. Top 100 Ranked Predictive Topics for Predicting 2-Year Mortality

Rank	Topic ID	Top 25 Probable Words
1	198	care hospice family comfort palliative dni dnr prn goal morphine cmo discussion dementia measure pain goc nurse status meet transition decline comfortable make continue hcp
2	424	status dementia unable mental baseline eye command nurse verbal alter hypernatremia open due poor lethargy intake lethargic care family respond question note history ass head
3	112	cholesterol ldl result test total blood compare hdl bad normal function triglyceride good hemoglobin medical perform history stable record increase decrease remain question dear letter
4	297	agitation agitate dementia seroquel delirium haldol continue zyprexa prn sitter behavior psych time medication trazodone sleep require refuse nurse increase staff behavioral night unable care
5	254	range normal detail test blood result function check glucose creatinine potassium kidney total calcium bun sodium electrolyte chloride carbon dioxide chemistry share receive letter manager
6	233	cancer lung metastatic disease chemotherapy oncology cycle radiation cell show chemo tumor carcinoma mass adenocarcinoma stage treatment metastasis node xrt scan carboplatin chest therapy week
7	21	pain therapy time treatment week increase level exercise physical functional report activity tissue visit hip management evaluation goal care progress strength muscle end base leave
8	361	date information case phone admit info referral status hospital salem care bed gender contact page birth confidential disch act update summary policy payor pdf home
9	190	result test letter question dear receive contact manager share normal hesitate blood function show report kidney apt sincerely street lynn stable electrolyte count cell detail
10	68	facility live assist nurse dementia rehab snf care transfer discharge staff term fall alf long unit back unable center skilled return admit hospital move baseline
11	397	nutrition intake weight protein day supplement goal meal clinical calorie kcal daily ensure diet continue malnutrition food energy loss estimate meet relate report kcals order

12	53	los para una con usted por tiene puede sus del mdico dolor como medicamentos sobre est esta son medicamento instrucciones llame tomar tambin durante cuidado
13	215	hospital general medication massachusetts management medicine associate fax internal phone pharmacy electronically transmit prescription prepare tab cardiology murray health end electronic north summary hold walgreens
14	321	knee pain leave joint osteoarthritis injection replacement medial surgery arthritis effusion total lateral bilateral motion range tear intact today degree tkr physical year negative swell
15	111	disorder axis problem visit mood anxiety coherent risk direct office treatment current pain exam status social individual symptom gaf mental type iii contact personality psychotherapy
16	213	lasix chf edema weight volume overload increase diuresis home daily heart failure sob admission fluid continue dose elevate bid bnp pulmonary exacerbation worsen day tte
17	13	skin lesion include extremity sun scalp discuss back perform exam papule upper dermatology face nevus today neck chest benign leave history complaint appear melanoma cancer
18	147	family care discuss meet time discussion make plan team decision son discus understand medical risk concern option goal continue speak life today home decline agree
19	453	care pain respiratory dementia extend gait edema weight gastrointestinal musculoskeletal med review wheeze clear erythema bun cre chronic complaint bowel egfr hct hgb constitutional facility
20	2	pain back leg tylenol leave neck low day lower side muscle tenderness week worse ibuprofen walk symptom worsen area numbness improve ago weakness report spine
21	30	test cholesterol blood follow function laboratory phone recent normal office dear range medicine parkman kidney call optimal question write study sincerely boston count mdima good
22	281	time visit pain therapy treatment week exercise report goal evaluation decrease demonstrate minute verbal end care hip hep start provide level physical functional date program
23	394	appointment schedule call reschedule cancel office show miss care appt time make letter patient day today contact prior dear physician health clinic follow cancellation hour

© 2019 Wang L et al. *JAMA Network Open*

24	403	care respiratory extend hct bun hgb cre wbc glu plt egfr rbc gastrointestinal musculoskeletal edema mchc mch mcv dementia med review clear constitutional bowel pain
25	196	status mental alter confusion baseline acute confuse head delirium admission change family improve time unable note negative infection orient recent find hospital dementia worsen due
26	292	care home call service icmp vna case manager visit plan nurse update pcp report follow speak program mgh management week referral contact today continue time
27	369	day feel week take today time visit medication back stop start make month tell med night state think issue good pill problem check work ask
28	116	dementia alzheimer hallucination seroquel day continue behavioral increase body care namenda lewy disease dose disturbance sleep family memantine quetiapine agitation night medication donepezil aricept start
29	174	pressure blood normal edema pulse weight clear chest murmur today year regular daily heart extremity pound continue abdomen hypertension month followup rate show cardiac exam
30	360	care extend pain dementia continue facility progress illness complaint present psych today gait hcp htn disorder respiratory edema rosewood cardiac sob due chronic cough admit
31	266	today colonoscopy screen year lipid repeat check normal continue visit due mammogram exam exercise breast weight pap ldl issue cancer health work htn month age
32	216	tablet day tab bid capsule qhs prn direct acid hcl unit release vitamin visit tid multivitamin sodium present aspirin calcium extend qpm illness mcg month
33	155	assist sit stand train bed gait mobility balance min physical session therapy therapeutic function home continue treatment transfer activity bill minute care ctg chair inpatient
34	184	daughter home family dementia care mother life report day time state today speak year concern live female week dtr bring law past granddaughter house ago
35	38	void symptom normal urinary prostate bladder mass score psa frequency nocturia time urgency incontinence urology today show stream year flow problem empty scrotal rectal scrotum

© 2019 Wang L et al. *JAMA Network Open*

36	102	subject message call refill original prescription reply forward NAME NAME phone number speak NAME med pharmacy request NAME NAME NAME vna fwd desk NAME NAME
37	285	swallow speech oral slp aspiration liquid cough language diet thin evaluation note voice adequate report present reduce disorder clear difficulty mgh solid baseline exam dysphagia
38	407	normal exam include find time pulse sit history note interpretation pain resp status extremity respiratory head physician record emergency department review rate newton inspection drug
39	158	bed continue shift progress nurse oob assist rehab pain time chair note urine today order skin place alarm remain monitor sit precaution deny overnight intact
40	76	pain lumbar leave extremity injection lower back bilateral spine physical negative review bilaterally therapy deny symptom leg history tenderness low normal joint intact muscle walk
41	448	pulse normal pain tenderness motion negative resp time note pmhx full range chest evidence intact equal year extremity date swell injury status redness sound history
42	113	date information phone case admit referral status info hospital bed gender contact care update page application properly dispose confidential retrieve birth act disch policy pdf
43	251	visit street boston hospital dear date sincerely woman question today brigham clinic hesitate care francis refer evaluation forward call number birth portion suite relevant pleasure
44	1	cough throat day fever symptom sinus congestion sore clear chest nasal week pain uri wheeze nose ear viral chill productive feel infection cold worsen bronchitis
45	382	wind ulcer dress pressure skin care change stage area sacral heal apply heel bed decubitus buttock leave coccyx drainage dry tissue cover nurse open mepilex
46	71	liquid swallow diet aspiration dysphagia thick puree slp nectar solid thin speech continue soft consistency intake eat food meal advance family oral recommend cough small
47	178	swallow slp liquid aspiration oral speech thin dysphagia solid diet puree language cough consistency thick nectar trial note family education evaluation today present time status
48	456	dialysis renal esrd fistula end stage hemodialysis disease nephrocaps sevelamer leave arm unit access graft acetate avf alfa catheter anemia failure labetalol mwf vein thrill

© 2019 Wang L et al. *JAMA Network Open*

49	497	progress hospitalization adult absence risk continue pediatric fall actual sign discharge infection symptom condition pressure pain imbalance demonstrate ulcer monitor intake fluid minimize electrolyte impair
50	379	pulse daily resp discharge normal physician time status sign salem care acute day hospital lpm mon date history primary year pmhx medical pain admission rate
51	122	aspiration pneumonia pna cough respiratory continue cxr recurrent risk secretion home sit antibiotic sputum airway admission cefepime due suction slp chest opacity hypoxia pneumonitis status
52	132	daily discharge hospital physician sign day status care admission medical blood show time shore primary north date room center admit salem electronically birth bedtime job
53	60	foot toe nail leave care shoe great pain skin toenail present note podiatry hallux plantar pulse lesion bilaterally bilateral infection visit month dpm painful diabetic
54	5	continue progress home hour intake output hcc monitor bwh spo mmhg hold data file medicine dvt net gross summary admission today cont read ppx encounter
55	75	consult history attend plan mgh find medicine htn review present admission resident admit care team exam discuss negative agree note pager include dementia obtain prior
56	162	sit bed mobility stand home assist function balance gait pac score train ctg min mod supine chair unable independent evaluation modifier base history fall wnl
57	39	today visit pain abd stable continue edema rrr ext nad lung htn soft gen follow heent chest weight clear neck sob month cta check deny
58	99	husband dementia home unable care female due wheelchair history caregiver family baseline report year time present obtain woman caretaker nurse aide question note alzheimer health
59	54	feel time work anxiety continue report depression stress mood week friend therapy think issue discuss sleep make talk thing good life depress support anxious activity
60	378	fever admission wbc cxr home elevate sepsis culture cefepime infection lactate continue blood baseline leukocytosis hold negative vanc hypotension fluid ivf urine low set source
61	459	visit minute pain exercise stand today session min program home hip total continue level stretch step report treatment good sit therapy education caregiver hep understand

© 2019 Wang L et al. *JAMA Network Open*

62	323	referral crms provider reason phone department assign detail insurance clinical urgency document comment flag user preceptor mgh system require pcp request campus contact primary question
63	300	shock sepsis transfer icu micu hypotension septic failure set pressor continue fluid require improve respiratory aki renal line start status admission floor volume acute blood
64	9	memory cognitive loss year drive visit difficulty term month husband impairment short continue decline mild recall disorder issue minute problem remember test time day forget
65	395	care extend pain due fall dementia cont respiratory gastrointestinal rehab musculoskeletal constitutional edema facility progress dnr skin anemia complaint illness present hcp hct neurologic uti
66	212	cholesterol negative report exam ldl desirable screen hdl triglyceride smoke tsh result influenza monitor status hemoglobin hgb hematocrit gfr hct colonoscopy hba estimate smoker health
67	246	respiratory bipap failure pulmonary oxygen hypoxia sit icu edema status transfer lasix distress require improve acute hypoxemia nasal abg home time wean breathe admission increase
68	256	disease chronic artery coronary heart hypertension renal diabetes failure peripheral hyperlipidemia kidney mellitus disorder pulmonary reflux vascular congestive gastroesophageal impairment obstructive anemia type stenosis cad
69	206	normal time note sit review pulse status history interpretation inspection pain physician resp skin respiratory record newton deny sign drug eye route chest nontender tenderness
70	396	call phone today state speak appt lab leave office result vna back tomorrow week nurse pcp report message advise return visit schedule subject plan check
71	398	year die age sister history brother life mother child father ago work smoke cancer live retire alcohol son daughter drink marry past drug month quit
72	260	pain chest ekg cardiac negative breath shortness symptom stress test normal episode discomfort report resolve heart leave troponin sob pressure deny nausea atypical side radiate
73	414	normal exam include find time pulse history note sit pain interpretation extremity respiratory head resp status physician department rate review motion inspection range orient newton

© 2019 Wang L et al. *JAMA Network Open*

74	393	skin lesion leave scar back scalp upper biopsy review include bcc scc neck consent sun papule shave wind procedure obtain face chest bleed extremity discuss
75	486	pulse negative normal pain pmhx resp appreciate time note date abdomen acute tenderness year sign extremity respiratory status hpi constitutional salem sound neuro cardiovascular deny
76	4	average memory test function difficulty score report recall performance cognitive word evaluation range learn low visual task impair neuropsychological error attention total verbal time high
77	93	pain stable discharge tolerate pod medication diet control operative post surgery incision transfer void procedure home hour condition oxycodone detail ambulate remain day hospital undergo
78	353	vaccine influenza injection pneumococcal flu order date dose receive administer shoot visit immunization health lot trivalent vaccination today preservative acupuncture month formulation high unspecified exp
79	161	intubate vent airway goal day continue tube fio cmh respiratory care line rate icu ett cpap propofol eye open neuro command wean remain peep intubation
80	284	osteoporosis calcium bone vitamin fracture score density hip bmd year spine fosamax total alendronate hyperparathyroidism hypercalcemia date osteopenia vit risk pth neck reclast endocrine age
81	51	failure heart lasix congestive chf chronic edema diastolic weight furosemide daily increase acute day breath shortness dose torsemide renal disease pulmonary bnp hypertension systolic cardiac
82	450	dizziness vertigo feel symptom meclizine head dizzy stand vestibular report positional sit gait test time lightheadedness headache day walk spin episode fall felt balance nystagmus
83	40	copd home pulmonary chronic exacerbation oxygen breath obstructive wheeze day disease prednisone shortness lung continue sob cough neb respiratory albuterol sit nebulizer smoke steroid breathe
84	240	date information care home phone admit referral hospital status case gender contact service update page properly application retrieve dispose confidential disch summary birth act policy
85	383	uti urinary infection urine tract day culture antibiotic treat recurrent cipro utis fever ceftriaxone bactrim symptom positive start coli frequency ciprofloxacin dysuria result bacteria back

© 2019 Wang L et al. *JAMA Network Open*

86	295	delirium continue agitation psychiatry prn consult haldol follow question seroquel think page time daily unable ass medication bed sleep qtc haloperidol current avoid qhs psych
87	121	picc catheter procedure line placement sterile site lumen access vein remove insertion dress length perform insert tip flush leave obtain arm consent sheath needle central
88	386	tablet take north mouth shore med transfer metal process date group daily physician prefer provider salem department birth nsp english dept phone language danvers address
89	268	normal negative cardiovascular constitutional skin sound chest exhibit pain neurological breath musculoskeletal respiratory abdominal positive place eye neck person orient hent time tenderness pulmonary psychiatric
90	106	coumadin atrial fibrillation inr warfarin afib digoxin metoprolol irregular control rate heart chronic chf fib lasix failure paroxysmal continue anticoagulation dose congestive daily irregularly furosemide
91	14	surgery anesthesia pre procedure risk general cardiac history date deny medication mgh surgical morning wnl schedule consent prior day sip water surgeon medical operative year
92	151	chelsea mgh subject referral phone call everett request fax message ave eileen evaluation nurse original form care appt contact vna refer nancy office lewis triage
93	3	procedure injection pain needle consent day site medication blood time today inject lidocaine prior skin ice appointment post instruction hold apply include hour sign prepped
94	81	cont neg med pain due eval visit past sxs htn stable appt sob rec neuro edema take mild today clear wnl change bilat prior decline
95	180	date status general review reaction bpm cardiac normal pulse chest rate lab massachusetts bruit recent edema hospital year electronically mmhg smoke weight follow document hypertension
96	460	blood pressure hypertension amlodipine lisinopril continue medication home htn daily control hypertensive dose elevate losartan low check increase hctz labetalol take atenolol high start monitor
97	148	today document street error escription tel recognition followup hill year chestnut voice excuse boston woman francis software brigham boylston medical visit month review transcription appear
98	443	drain abscess drainage collection fluid tube cholecystitis surgery placement percutaneous cholecystostomy abdominal chole catheter

© 2019 Wang L et al. *JAMA Network Open*

		flush perc place antibiotic output flagyl abdomen site surgical radiology image
99	228	incontinence vaginal urinary prolapse bladder pessary normal pelvic time visit estrogen void urge cream urine premarin vagina urgency cystocele frequency ring estring uterine floor urinate
100	199	anxiety depression continue increase medication dose disorder sleep trazodone start mirtazapine daily clonazepam insomnia wellbutrin day mood remeron bupropion qhs depressive klonopin lorazepam sertraline effect

NAME: we replace any mention of human names with the 'NAME'.

© 2019 Wang L et al. *JAMA Network Open*

eTable 3. Top 100 Ranked Predictive Topics for Predicting 1-Year Mortality

Rank	Topic ID	Top 25 Probable Words
1	198	care hospice family comfort palliative dni dnr prn goal morphine cmo discussion dementia measure pain goc nurse status meet transition decline comfortable make continue hcp
2	424	status dementia unable mental baseline eye command nurse verbal alter hypernatremia open due poor lethargy intake lethargic care family respond question note history ass head
3	254	range normal detail test blood result function check glucose creatinine potassium kidney total calcium bun sodium electrolyte chloride carbon dioxide chemistry share receive letter manager
4	112	cholesterol ldl result test total blood compare hdl bad normal function triglyceride good hemoglobin medical perform history stable record increase decrease remain question dear letter
5	190	result test letter question dear receive contact manager share normal hesitate blood function show report kidney apt sincerely street lynn stable electrolyte count cell detail
6	216	tablet day tab bid capsule qhs prn direct acid hcl unit release vitamin visit tid multivitamin sodium present aspirin calcium extend qpm illness mcg month
7	147	family care discuss meet time discussion make plan team decision son discus understand medical risk concern option goal continue speak life today home decline agree
8	206	normal time note sit review pulse status history interpretation inspection pain physician resp skin respiratory record newton deny sign drug eye route chest nontender tenderness
9	297	agitation agitate dementia seroquel delirium haldol continue zyprexa prn sitter behavior psych time medication trazodone sleep require refuse nurse increase staff behavioral night unable care
10	215	hospital general medication massachusetts management medicine associate fax internal phone pharmacy electronically transmit prescription prepare tab cardiology murray health end electronic north summary hold walgreens
11	68	facility live assist nurse dementia rehab snf care transfer discharge staff term fall alf long unit back unable center skilled return admit hospital move baseline

© 2019 Wang L et al. *JAMA Network Open*

12	30	test cholesterol blood follow function laboratory phone recent normal office dear range medicine parkman kidney call optimal question write study sincerely boston count mdima good
13	21	pain therapy time treatment week increase level exercise physical functional report activity tissue visit hip management evaluation goal care progress strength muscle end base leave
14	53	los para una con usted por tiene puede sus del mdico dolor como medicamentos sobre est esta son medicamento instrucciones llame tomar tambin durante cuidado
15	361	date information case phone admit info referral status hospital salem care bed gender contact page birth confidential disch act update summary policy payor pdf home
16	329	continue progress rate output intake hour today spo urine monitor total overnight shift nurse event net respiratory day min plan mmhg team bed soft temp
17	111	disorder axis problem visit mood anxiety coherent risk direct office treatment current pain exam status social individual symptom gaf mental type iii contact personality psychotherapy
18	71	liquid swallow diet aspiration dysphagia thick puree slp nectar solid thin speech continue soft consistency intake eat food meal advance family oral recommend cough small
19	233	cancer lung metastatic disease chemotherapy oncology cycle radiation cell show chemo tumor carcinoma mass adenocarcinoma stage treatment metastasis node xrt scan carboplatin chest therapy week
20	397	nutrition intake weight protein day supplement goal meal clinical calorie kcal daily ensure diet continue malnutrition food energy loss estimate meet relate report kcals order
21	122	aspiration pneumonia pna cough respiratory continue cxr recurrent risk secretion home sit antibiotic sputum airway admission cefepime due suction slp chest opacity hypoxia pneumonitis status
22	360	care extend pain dementia continue facility progress illness complaint present psych today gait hcp htn disorder respiratory edema rosewood cardiac sob due chronic cough admit
23	285	swallow speech oral slp aspiration liquid cough language diet thin evaluation note voice adequate report present reduce disorder clear difficulty mgh solid baseline exam dysphagia

© 2019 Wang L et al. *JAMA Network Open*

24	323	referral crms provider reason phone department assign detail insurance clinical urgency document comment flag user preceptor mgh system require pcp request campus contact primary question
25	180	date status general review reaction bpm cardiac normal pulse chest rate lab massachusetts bruit recent edema hospital year electronically mmhg smoke weight follow document hypertension
26	174	pressure blood normal edema pulse weight clear chest murmur today year regular daily heart extremity pound continue abdomen hypertension month followup rate show cardiac exam
27	453	care pain respiratory dementia extend gait edema weight gastrointestinal musculoskeletal med review wheeze clear erythema bun cre chronic complaint bowel egfr hct hgb constitutional facility
28	435	medication discharge take start list mouth dose day hospital page massachusetts general dob time pdf continue pmpatient allergic reaction instruction report diagnosis content provider original
29	196	status mental alter confusion baseline acute confuse head delirium admission change family improve time unable note negative infection orient recent find hospital dementia worsen due
30	341	sit stand assist home bed level cue ambulation supine assistance device mobility transfer walker progress chair therapy roll clinical strength impair fall pain observation verbal
31	132	daily discharge hospital physician sign day status care admission medical blood show time shore primary north date room center admit salem electronically birth bedtime job
32	130	time tablet medication mouth home result care discharge test question total pending daily hospitalization problem rate admission health dose day contact detail frequency hospital take
33	369	day feel week take today time visit medication back stop start make month tell med night state think issue good pill problem check work ask
34	322	general today review weight pressure blood reaction electronically status massachusetts document return bpm associate sign pulse hospital final medicine appearance medication internal mmhg year risk
35	456	dialysis renal esrd fistula end stage hemodialysis disease nephrocaps sevelamer leave arm unit access graft acetate avf alfa catheter anemia failure labetalol mwf vein thrill

© 2019 Wang L et al. *JAMA Network Open*

36	94	time balance gait treatment therapy sec stand exercise hip report walk fall physical step min visit pain week knee foot tandem second sit train increase
37	155	assist sit stand train bed gait mobility balance min physical session therapy therapeutic function home continue treatment transfer activity bill minute care ctg chair inpatient
38	44	sit assist stand bed cue mobility transfer min chair balance gait walker continue train session supine max mod activity rehab require ambulation step roll supervision
39	470	normal mass clear tenderness neck skin chest pulse murmur lesion abdomen edema adenopathy sound node bruit extremity supple soft negative cervical rub gallop thyroid intact
40	2	pain back leg tylenol leave neck low day lower side muscle tenderness week worse ibuprofen walk symptom worsen area numbness improve ago weakness report spine
41	148	today document street error escription tel recognition followup hill year chestnut voice excuse boston woman francis software brigham boylston medical visit month review transcription appear
42	260	pain chest ekg cardiac negative breath shortness symptom stress test normal episode discomfort report resolve heart leave troponin sob pressure deny nausea atypical side radiate
43	403	care respiratory extend hct bun hgb cre wbc glu plt egfr rbc gastrointestinal musculoskeletal edema mchc mch mcv dementia med review clear constitutional bowel pain
44	281	time visit pain therapy treatment week exercise report goal evaluation decrease demonstrate minute verbal end care hip hep start provide level physical functional date program
45	171	pancreatic pancreas mass cancer duct lesion ipmn head neoplasm whipple show adenocarcinoma cyst scan cystic abdominal find tail mucinous pain gemcitabine tumor weight body mrcp
46	99	husband dementia home unable care female due wheelchair history caregiver family baseline report year time present obtain woman caretaker nurse aide question note alzheimer health
47	172	brain mri leave meningioma frontal lesion resection mass tumor lobe craniotomy temporal stable enhance enhancement show increase image find prior neuro parietal edema post intracranial
48	102	subject message call refill original prescription reply forward NAME NAME phone number speak NAME med pharmacy request NAME NAME NAME vna fwd desk NAME NAME

© 2019 Wang L et al. *JAMA Network Open*

49	205	unknown tablet report pharmacy mouth daily total capsule day visit current time file prior administer outpatient facility prescription medication nightly unit need encounter vitamin aspirin
50	78	normal pain tenderness back chest adenopathy weight wheeze problem history blood complaint bruit abdomen joint pulse murmur palpitation respiratory ear neurologic chief pressure fever medication
51	390	range normal detail test neg blood result check auto negative function total wbc cell glucose bilirubin protein hct hgb creatinine plt count chemistry rbc mcv
52	451	normal limit component notable abnormal present negative panel glucose file cbc basic observation history metabolic review temporal lab find emergency result resp spo status differential
53	41	care risk home health icmp issue plan screen medical multiple daughter date service mgh program advance management follow utilization status independent intervention directive manager life
54	118	order time complete pulse normal edms drug resp route note follow sign negative ekg nurse status abdomen exam result response rmm year medication temp medical
55	32	dose discharge admission bid diagnosis vaccine prn study lab admit unit sodium status code mgh time floor treatment dob follow life number sustain principal qhs
56	466	emergency rate year date extremity room time dictate blood history department clear report sign bilaterally pressure deny status nontender pain regular medical chest state distress
57	293	lad rca stent coronary artery leave cath lesion stenosis mid proximal cardiac catheterization pci lcx distal vessel procedure cad circumflex main patent angiography balloon disease
58	417	stroke leave side infarct mca acute weakness neurology facial tpa command neuro mri head arm show htn droop motor start speech gaze cta exam aphasia
59	113	date information phone case admit referral status info hospital bed gender contact care update page application properly dispose confidential retrieve birth act disch policy pdf
60	120	general hospital sign massachusetts status document electronically final medicine associate internal telephone call update chart end north pager state NAME NAME subject health narrative today
61	131	son home life live family report house care state day fall visit time concern apartment walk service meal dementia safety deny walker health move take

© 2019 Wang L et al. *JAMA Network Open*

62	213	lasix chf edema weight volume overload increase diuresis home daily heart failure sob admission fluid continue dose elevate bid bnp pulmonary exacerbation worsen day tte
63	431	subject call message phone original reply NAME NAME NAME desk speak fadi forward NAME NAME fwd NAME NAME NAME NAME NAME NAME NAME NAME back
64	221	chest lung effusion pleural leave pulmonary opacity pneumonia view atelectasis edema report unchanged pneumothorax heart mild bilateral lobe evidence lateral lower mediastinum comparison cxr small
65	364	carotid stenosis artery leave ica internal cea endarterectomy disease vertebral vascular note mild ultrasound moderate bilateral severe flow proximal plaque systolic subclavian distal external significant
66	175	culture infection vancomycin antibiotic bacteremia blood picc fever mrsa continue line disease infectious negative grow staph week gram abx positive mssa vanco abscess endocarditis dose
67	394	appointment schedule call reschedule cancel office show miss care appt time make letter patient day today contact prior dear physician health clinic follow cancellation hour
68	142	partner healthcare home determine take NAME NAME NAME unscheduled NAME NAME provider otr jam daily mouth NANE NAME department NAME NAME prefer NAME NAME NAME
69	490	seizure keppra disorder bid eeg neurology activity level levetiracetam dilantin continue episode depakote epilepsy increase dose day phenytoin status medication time lamictal brain neuro home
70	95	medicine provider care caregiver instruction visit follow primary pain health healthcare make include drink feel test information blood emergency decrease prevent call treatment breathe cause
71	124	nbsp find significant plan general date intact abdomen head neck hpi time chest respiratory differential normal skin sign edis heart cardiovascular attend status emergency eye
72	404	negative normal pain change chest positive skin cardiovascular constitutional eye abdominal exhibit neurological neck musculoskeletal hent respiratory tenderness breath sound ear swell psychiatric present gastrointestinal
73	173	instruction visit care provider follow medicine emergency perform test primary health date department discharge final find important appointment treatment review report receive bwh talk make

© 2019 Wang L et al. *JAMA Network Open*

74	178	swallow slp liquid aspiration oral speech thin dysphagia solid diet puree language cough consistency thick nectar trial note family education evaluation today present time status
75	18	bladder hematuria cystoscopy cancer tumor urology procedure normal carcinoma urine bcg cell urethra cytology turbt wall grade gross transitional resection mass papillary urothelial lesion ureteral
76	294	fracture injury collar hematoma trauma fall leave spine consult facial head surgery cervical orbital stable traumatic face bone transfer displace time sinus soft bilateral laceration
77	389	count platelet hematology thrombocytopenia leukemia marrow blood cll anemia bone cell transfusion bleed chronic show week cbc today time plt continue wbc year start syndrome
78	385	function functional activity base history evaluation occupational adl analysis result assist performance level care task require cognitive independent interview cue management include home supervision visual
79	497	progress hospitalization adult absence risk continue pediatric fall actual sign discharge infection symptom condition pressure pain imbalance demonstrate ulcer monitor intake fluid minimize electrolyte impair
80	133	group session skill therapy goal treatment plan time participation cop focus progress continue support attend management day engage minute cognitive psychotherapy identify think participate active
81	436	mother cancer relative maternal father disease paternal sister grandmother aunt brother hypertension diabetes artery coronary breast uncle grandfather type alzheimer colon age family mellitus negative
82	225	file present find review consult history negative pain head plan skin temporal spo order respiratory neck medical emergency resp status intact temp pulse normal chest
83	460	blood pressure hypertension amlodipine lisinopril continue medication home htn daily control hypertensive dose elevate losartan low check increase hctz labetalol take atenolol high start monitor
84	237	dana cancer farber institute center oncology service woman department brigham bwh medication brookline infusion address therapy ave boston yawkey dfci appointment tablet floor laboratory radiation

© 2019 Wang L et al. *JAMA Network Open*

85	321	knee pain leave joint osteoarthritis injection replacement medial surgery arthritis effusion total lateral bilateral motion range tear intact today degree tkr physical year negative swell
86	218	mouth change day medication discharge time admission need home hour final dob summary page telephone phone sodium address frequency list dose skin hcl multivitamin plan
87	469	daily history date status add time newton hospital admission patient admit blood year adm room emergency attend pulse physician show extremity pressure rate wellesley care
88	407	normal exam include find time pulse sit history note interpretation pain resp status extremity respiratory head physician record emergency department review rate newton inspection drug
89	28	stroke weakness mri speech tia facial neurology head leave symptom acute side droop episode difficulty brain slur neuro transient show ischemic mra find aspirin neck
90	212	cholesterol negative report exam ldl desirable screen hdl triglyceride smoke tsh result influenza monitor status hemoglobin hgb hematocrit gfr hct colonoscopy hba estimate smoker health
91	347	mouth day care discharge time medicine admission change phone home instruction doctor dob need date independent hospital address activity referral question hour applicable appointment service
92	279	partner gateway account day support information take org patientgateway activation email call log make message effort team business phone visit activate tablet code www expire
93	247	mouth change day discharge admission medication home time final dob page summary need telephone list phone hour frequency address plan dfci care mrn dose find
94	195	spanish take bwh center plain jamaica health preferido idioma hispanic total del personal fecha mdico departamento direccin dept southern visit pain alergias partir raza latino
95	493	artery leave stenosis carotid vertebral mra neck intracranial internal posterior cta segment aneurysm evidence mild vessel significant narrow cerebral proximal origin moderate bilateral mri basilar
96	161	intubate vent airway goal day continue tube fio cmh respiratory care line rate icu ett cpap propofol eye open neuro command wean remain peep intubation
97	214	find significant plan intact hpi abdomen head nbsp respiratory neck chest differential agent normal skin general cardiovascular heart eye date review time ent take musculoskeletal

© 2019 Wang L et al. *JAMA Network Open*

98	381	bleed blood rectal hemorrhoid stool colonoscopy red epistaxis rectum bright diverticulosis episode brbpr stable note pack exam diverticular internal active lower clot small pain hct
99	13	skin lesion include extremity sun scalp discuss back perform exam papule upper dermatology face nevus today neck chest benign leave history complaint appear melanoma cancer
100	51	failure heart lasix congestive chf chronic edema diastolic weight furosemide daily increase acute day breath shortness dose torsemide renal disease pulmonary bnp hypertension systolic cardiac

NAME: we replace any mention of human names with the 'NAME'.

© 2019 Wang L et al. *JAMA Network Open*

eTable 4. Top 100 Ranked Predictive Topics for Predicting 6-Month Mortality

Ranks	Topic ID	Top 25 Probable Words
1	198	care hospice family comfort palliative dni dnr prn goal morphine cmo discussion dementia measure pain goc nurse status meet transition decline comfortable make continue hcp
2	424	status dementia unable mental baseline eye command nurse verbal alter hypernatremia open due poor lethargy intake lethargic care family respond question note history ass head
3	254	range normal detail test blood result function check glucose creatinine potassium kidney total calcium bun sodium electrolyte chloride carbon dioxide chemistry share receive letter manager
4	112	cholesterol ldl result test total blood compare hdl bad normal function triglyceride good hemoglobin medical perform history stable record increase decrease remain question dear letter
5	190	result test letter question dear receive contact manager share normal hesitate blood function show report kidney apt sincerely street lynn stable electrolyte count cell detail
6	206	normal time note sit review pulse status history interpretation inspection pain physician resp skin respiratory record newton deny sign drug eye route chest nontender tenderness
7	147	family care discuss meet time discussion make plan team decision son discus understand medical risk concern option goal continue speak life today home decline agree
8	215	hospital general medication massachusetts management medicine associate fax internal phone pharmacy electronically transmit prescription prepare tab cardiology murray health end electronic north summary hold walgreens
9	297	agitation agitate dementia seroquel delirium haldol continue zyprexa prn sitter behavior psych time medication trazodone sleep require refuse nurse increase staff behavioral night unable care
10	397	nutrition intake weight protein day supplement goal meal clinical calorie kcal daily ensure diet continue malnutrition food energy loss estimate meet relate report kcals order
11	497	progress hospitalization adult absence risk continue pediatric fall actual sign discharge infection symptom condition pressure pain imbalance demonstrate ulcer monitor intake fluid minimize electrolyte impair

12	246	respiratory bipap failure pulmonary oxygen hypoxia sit icu edema status transfer lasix distress require improve acute hypoxemia nasal abg home time wean breathe admission increase
13	300	shock sepsis transfer icu micu hypotension septic failure set pressor continue fluid require improve respiratory aki renal line start status admission floor volume acute blood
14	178	swallow slp liquid aspiration oral speech thin dysphagia solid diet puree language cough consistency thick nectar trial note family education evaluation today present time status
15	71	liquid swallow diet aspiration dysphagia thick puree slp nectar solid thin speech continue soft consistency intake eat food meal advance family oral recommend cough small
16	174	pressure blood normal edema pulse weight clear chest murmur today year regular daily heart extremity pound continue abdomen hypertension month followup rate show cardiac exam
17	216	tablet day tab bid capsule qhs prn direct acid hcl unit release vitamin visit tid multivitamin sodium present aspirin calcium extend qpm illness mcg month
18	161	intubate vent airway goal day continue tube fio cmh respiratory care line rate icu ett cpap propofol eye open neuro command wean remain peep intubation
19	68	facility live assist nurse dementia rehab snf care transfer discharge staff term fall alf long unit back unable center skilled return admit hospital move baseline
20	453	care pain respiratory dementia extend gait edema weight gastrointestinal musculoskeletal med review wheeze clear erythema bun cre chronic complaint bowel egfr hct hgb constitutional facility
21	360	care extend pain dementia continue facility progress illness complaint present psych today gait hcp htn disorder respiratory edema rosewood cardiac sob due chronic cough admit
22	30	test cholesterol blood follow function laboratory phone recent normal office dear range medicine NAME kidney call optimal question write study sincerely boston count mdima good
23	329	continue progress rate output intake hour today spo urine monitor total overnight shift nurse event net respiratory day min plan mmhg team bed soft temp

24	416	transfer arrest icu start osh ccu cardiac intubate receive time floor find arrival initially gtt admission note micu require family show hypotension unresponsive intubation prior
25	53	los para una con usted por tiene puede sus del mdico dolor como medicamentos sobre est esta son medicamento instrucciones llame tomar tambin durante cuidado
26	173	instruction visit care provider follow medicine emergency perform test primary health date department discharge final find important appointment treatment review report receive bwh talk make
27	75	consult history attend plan mgh find medicine htn review present admission resident admit care team exam discuss negative agree note pager include dementia obtain prior
28	122	aspiration pneumonia pna cough respiratory continue cxr recurrent risk secretion home sit antibiotic sputum airway admission cefepime due suction slp chest opacity hypoxia pneumonitis status
29	403	care respiratory extend hct bun hgb cre wbc glu plt egfr rbc gastrointestinal musculoskeletal edema mchc mch mcv dementia med review clear constitutional bowel pain
30	121	picc catheter procedure line placement sterile site lumen access vein remove insertion dress length perform insert tip flush leave obtain arm consent sheath needle central
31	378	fever admission wbc cxr home elevate sepsis culture cefepime infection lactate continue blood baseline leukocytosis hold negative vanc hypotension fluid ivf urine low set source
32	158	bed continue shift progress nurse oob assist rehab pain time chair note urine today order skin place alarm remain monitor sit precaution deny overnight intact
33	394	appointment schedule call reschedule cancel office show miss care appt time make letter patient day today contact prior dear physician health clinic follow cancellation hour
34	486	pulse negative normal pain pmhx resp appreciate time note date abdomen acute tenderness year sign extremity respiratory status hpi constitutional salem sound neuro cardiovascular deny
35	5	continue progress home hour intake output hcc monitor bwh spo mmhg hold data file medicine dvt net gross summary admission today cont read ppx encounter
36	262	tube fee peg feed hour day placement gastrostomy water nutrition bolus flush goal time gastric free osmolite protein site continue place gtube npo rate continuous

© 2019 Wang L et al. *JAMA Network Open*

37	196	status mental alter confusion baseline acute confuse head delirium admission change family improve time unable note negative infection orient recent find hospital dementia worsen due
38	2	pain back leg tylenol leave neck low day lower side muscle tenderness week worse ibuprofen walk symptom worsen area numbness improve ago weakness report spine
39	369	day feel week take today time visit medication back stop start make month tell med night state think issue good pill problem check work ask
40	155	assist sit stand train bed gait mobility balance min physical session therapy therapeutic function home continue treatment transfer activity bill minute care ctg chair inpatient
41	111	disorder axis problem visit mood anxiety coherent risk direct office treatment current pain exam status social individual symptom gaf mental type iii contact personality psychotherapy
42	396	call phone today state speak appt lab leave office result vna back tomorrow week nurse pcp report message advise return visit schedule subject plan check
43	298	fracture pain compression back spine vertebral lumbar thoracic fall osteoporosis chronic body acute kyphoplasty lower mri low vertebra brace severe multiple tylenol deformity oxycodone show
44	361	date information case phone admit info referral status hospital salem care bed gender contact page birth confidential disch act update summary policy payor pdf home
45	459	visit minute pain exercise stand today session min program home hip total continue level stretch step report treatment good sit therapy education caregiver hep understand
46	455	trach respiratory care tracheostomy airway secretion site tolerate progress status cuff continue assessment day suction cannula hospitalization vent aspiration cmh pressure failure dry clean condition
47	222	hemorrhage subdural hematoma sdh head leave neurosurgery sah stable keppra frontal repeat subarachnoid neuro find fall bleed iph transfer midline bilateral evacuation temporal hospital small
48	292	care home call service icmp vna case manager visit plan nurse update pcp report follow speak program mgh management week referral contact today continue time

© 2019 Wang L et al. *JAMA Network Open*

49	213	lasix chf edema weight volume overload increase diuresis home daily heart failure sob admission fluid continue dose elevate bid bnp pulmonary exacerbation worsen day tte
50	6	difficulty word speech leave hand progressive test language year include bilaterally atrophy find write normal movement ftd aphasia behavior time show read frontotemporal family slow
51	233	cancer lung metastatic disease chemotherapy oncology cycle radiation cell show chemo tumor carcinoma mass adenocarcinoma stage treatment metastasis node xrt scan carboplatin chest therapy week
52	273	care palliative home medical deny time pain life visit support goal symptom illness review plan family continue information history decline past month molst base management
53	354	progress hospitalization adult absence risk discharge condition day demonstrate impair fall infection skin pediatric actual level care pain symptom sign minimize wind nurse injury daily
54	39	today visit pain abd stable continue edema rrr ext nad lung htn soft gen follow heent chest weight clear neck sob month cta check deny
55	417	stroke leave side infarct mca acute weakness neurology facial tpa command neuro mri head arm show htn droop motor start speech gaze cta exam aphasia
56	251	visit street boston hospital dear date sincerely woman question today brigham clinic hesitate care francis refer evaluation forward call number birth portion suite relevant pleasure
57	118	order time complete pulse normal edms drug resp route note follow sign negative ekg nurse status abdomen exam result response rmm year medication temp medical
58	21	pain therapy time treatment week increase level exercise physical functional report activity tissue visit hip management evaluation goal care progress strength muscle end base leave
59	74	trach respiratory care tracheostomy failure chronic aspiration secretion site suction infection airway hour vent data pna recurrent progress seizure stable peg set acute cuff pressure
60	76	pain lumbar leave extremity injection lower back bilateral spine physical negative review bilaterally therapy deny symptom leg history tenderness low normal joint intact muscle walk

© 2019 Wang L et al. *JAMA Network Open*

61	323	referral crms provider reason phone department assign detail insurance clinical urgency document comment flag user preceptor mgh system require pcp request campus contact primary question
62	377	continue progress rate spo medicine team min mgh shift data max home hospital temp overnight intake bed prophylaxis urine nurse prn tcurrent hold result review
63	382	wind ulcer dress pressure skin care change stage area sacral heal apply heel bed decubitus buttock leave coccyx drainage dry tissue cover nurse open mepilex
64	113	date information phone case admit referral status info hospital bed gender contact care update page application properly dispose confidential retrieve birth act disch policy pdf
65	483	normal pulse negative pain time note pmhx date year sign extremity symptom tenderness resp female respiratory age status medical present abdomen discharge state physician documentation
66	60	foot toe nail leave care shoe great pain skin toenail present note podiatry hallux plantar pulse lesion bilaterally bilateral infection visit month dpm painful diabetic
67	463	tablet mouth total daily day time need capsule hour take nightly pain gram unit place acetaminophen sodium docusate colace tylenol senna constipation medication packet glycol
68	162	sit bed mobility stand home assist function balance gait pac score train ctg min mod supine chair unable independent evaluation modifier base history fall wnl
69	322	general today review weight pressure blood reaction electronically status massachusetts document return bpm associate sign pulse hospital final medicine appearance medication internal mmhg year risk
70	421	neg history stable low advise prior risk normal dose post recent evidence coley good mild sign issue age stop NAME fall status exam med evid
71	51	failure heart lasix congestive chf chronic edema diastolic weight furosemide daily increase acute day breath shortness dose torsemide renal disease pulmonary bnp hypertension systolic cardiac
72	128	negative urine wbc mmol plasma rbc glucose absolute protein bilirubin hpf lymph mono eos auto nitrite yellow blood gravity specific ketone clear urobilinogen total cell

© 2019 Wang L et al. *JAMA Network Open*

73	156	nbsp find date general plan time abdomen significant head intact hpi neck respiratory chest sign edis differential status normal emergency skin attend mgh heart cardiovascular
74	401	cognitive impairment visit prior moca mild continue concern dementia fall decline medication review history memory gait scott include good recommend increase mcginnis mood difficulty drive
75	47	spanish speak maria interpreter daughter family sanchez puerto rico carmen gonzalez day care jose language rodriguez health rivera boston clinic dominican manuel luis year republic
76	93	pain stable discharge tolerate pod medication diet control operative post surgery incision transfer void procedure home hour condition oxycodone detail ambulate remain day hospital undergo
77	341	sit stand assist home bed level cue ambulation supine assistance device mobility transfer walker progress chair therapy roll clinical strength impair fall pain observation verbal
78	450	dizziness vertigo feel symptom meclizine head dizzy stand vestibular report positional sit gait test time lightheadedness headache day walk spin episode fall felt balance nystagmus
79	445	tablet pain normal blood day weight visit pressure pulse present edema sign wheeze change abd neuro murmur tab illness bid reason rub stable gallop weakness
80	247	mouth change day discharge admission medication home time final dob page summary need telephone list phone hour frequency address plan dfci care mrn dose find
81	333	balance gait stand walk week fall step foot second cane increase report improve train turn time sec therapy sit home eye progress speed support stair
82	44	sit assist stand bed cue mobility transfer min chair balance gait walker continue train session supine max mod activity rehab require ambulation step roll supervision
83	425	pain neg negative repeat die mild normal show chest hypertension mri benign disease polyp colonoscopy nodule echo back stable chronic lung cyst renal osteoporosis elevate
84	311	spiritual spirituality outcome support care faith religion assessment service religious chaplain chaplaincy prayer consult express resource family inpatient illness visit catholic summary high emotional cop

© 2019 Wang L et al. *JAMA Network Open*

85	175	culture infection vancomycin antibiotic bacteremia blood picc fever mrsa continue line disease infectious negative grow staph week gram abx positive mssa vanco abscess endocarditis dose
86	240	date information care home phone admit referral hospital status case gender contact service update page properly application retrieve dispose confidential disch summary birth act policy
87	250	adrenal pituitary testosterone insufficiency endocrine normal dose cortisol hydrocortisone test level adenoma low prednisone note mri resection steroid tsh free endocrinology central replacement hypogonadism tumor
88	94	time balance gait treatment therapy sec stand exercise hip report walk fall physical step min visit pain week knee foot tandem second sit train increase
89	187	pain negative loss problem respiratory skin vision chest eye cardiovascular blood weight normal cough musculoskeletal change fever rash joint breath headache shortness gastrointestinal nausea palpitation
90	160	dialysis esrd renal access avf fistula today volume unit mwf continue schedule anemia tunnel hemodialysis clear end catheter leave skin daily stage phos soft rate
91	312	effusion pleural chest leave fluid pericardial thoracentesis side small lung cxr breath moderate large show bilateral decrease base pulmonary tube atelectasis drain increase sound empyema
92	148	today document street error escription tel recognition followup hill year chestnut voice excuse boston woman francis software brigham boylston medical visit month review transcription appear
93	423	food eat diet weight meal drink day low fruit vegetable high fat salt sodium milk snack exercise cup avoid water time meat juice intake add
94	420	swallow liquid aspiration pharyngeal thin residue dysphagia solid thick penetration bolus oral consistency study laryngeal nectar complete video tongue barium reduce cough clear speech slp
95	304	panel order metabolic complete cbc lab future stand bwh bwf dfci relevant basic nwh lipid check mgh today comprehensive tsh status date nsmc differential srh
96	40	copd home pulmonary chronic exacerbation oxygen breath obstructive wheeze day disease prednisone shortness lung continue sob cough neb respiratory albuterol sit nebulizer smoke steroid breathe

© 2019 Wang L et al. *JAMA Network Open*

97	81	cont neg med pain due eval visit past sxs htn stable appt sob rec neuro edema take mild today clear wnl change bilat prior decline
98	104	leave abdomen lesion image pelvis contrast normal small nodule measure adrenal lower calcification thicken report evidence node lobe kidney mass chest focal lymph wall tissue
99	217	deny pain fever chest nausea chill breath vomit shortness report abdominal symptom cough weakness headache diarrhea palpitation feel dizziness loss weight fatigue change dysuria sob
100	373	daily tab oral bid vitamin prn bedtime review aspirin unit qpm confirm reaction tablet multivitamin cap tid day status qam extend direct release mcg omeprazole

NAME: we replace any mention of human names with the 'NAME'.

© 2019 Wang L et al. *JAMA Network Open*
